# CT-Derived L3 Skeletal Muscle Index, Handgrip Strength, and Nutritional Behavior as Complementary Dimensions of Frailty in Older Oncology Inpatients: A Cross-Sectional Observational Study

**DOI:** 10.3390/nu18213550

**Published:** 2026-10-28

**Authors:** Sören Klaus Büntzel, Lea Büthe, Sabrina Sulzer, Jens Büntzel, Judith Büntzel

**Affiliations:** 1 Department of Radiotherapy and Radiation Oncology, Hannover Medical School, 30625 Hannover, Germany; 2 Department of Hematology and Medical Oncology, University Hospital, 37075 Göttingen, Germany; 3 Department of Gastroenterology, Gastrointestinal Oncology and Endocrinology, University Medical Center Göttingen, 37075 Göttingen, Germany; 4 Department of Otolaryngology, Head Neck Surgery, Südharz Hospital, 99734 Nordhausen, Germany; jens.buentzel@shk-ndh.de

**Keywords:** sarcopenia, frailty, geriatric oncology, skeletal muscle index, handgrip strength, nutritional behavior, nutritional assessment

## Abstract

**Background:** In older oncology patients, sarcopenia and frailty are highly prevalent but may reflect distinct vulnerability domains. Whether morphologic muscle loss, functional impairment, and nutritional behavior converge in this population remains unclear. We investigated whether CT-derived L3 skeletal muscle index, handgrip strength, G8-based frailty-related vulnerability, and self-reported nutritional behavior represent overlapping or distinct vulnerability dimensions in older oncology inpatients. **Methods:** This single-center substudy was derived from a prospectively collected screening cohort of hospitalized patients aged ≥65 years. Skeletal muscle index (SMI) was retrospectively quantified at the L3 level from routine CT scans acquired within ±1 month of screening, using sex-specific Prado cut-offs to classify CT-defined low skeletal muscle mass. Handgrip strength was measured by digital dynamometry and categorized using age- and sex-adjusted thresholds. Frailty was assessed with the G8 questionnaire, including item C on mobility. Nutritional behavior, comprising food avoidance, supplement use, and cancer-specific diets, was evaluated by structured self-report. Principal component analysis and correlation heat mapping were used to identify behavioral patterns. **Results:** Among 116 patients, including 68 with hematologic malignancies and 48 with solid tumors, CT-defined low muscle mass was present in 55.2% and was more frequent in men (*p* < 0.01). SMI was not associated with handgrip strength (rho = 0.03; *p* = 0.784). Reduced handgrip strength was associated with lower G8 scores (*p* < 0.001) and lower G8 item C mobility scores (*p* = 0.001). Self-reported nutritional behaviors were common and showed no significant association with CT-defined low skeletal muscle mass status. **Conclusions:** CT-derived muscle mass, muscle strength, and nutritional behavior appear to capture partly non-overlapping vulnerability dimensions in older oncology inpatients, supporting integrated functional and nutritional assessment.

## 1. Introduction

Sarcopenia, defined as a generalized and progressive loss of muscle mass and muscle strength, is a frequent and clinically relevant syndrome in older patients [1], particularly in the oncological setting [2,3]. With increasing age, the prevalence of sarcopenia rises substantially and is associated with higher mortality as well as loss of functional capacity in daily life, autonomy, and mobility [4,5]. This is of particular importance in an ageing oncology patient population, since functional status and mobility are key determinants of treatment choice, tolerability, and quality of life [2,3].

For the quantification of muscle mass, imaging methods are considered the reference standard: the assessment of muscle mass using dual-energy X-ray absorptiometry (DEXA) or the calculation of the skeletal muscle index (SMI) from computed tomography slices at the level of L3 are established approaches for assessing skeletal muscle quantity [6,7]. In oncological routine care, CT examinations are often performed as part of standard staging, making L3-based SMI measurements a pragmatic surrogate marker of muscle mass [6,8]. At the same time, assessment of muscle mass alone is not sufficient, because sarcopenia by definition comprises both structural and functional components, and functional impairments in everyday life are often the clinically more relevant aspect [1,9].

Geriatric assessment is recommended to identify age-associated vulnerabilities in older adults receiving systemic cancer therapy, including impairments that may not be captured by routine oncological assessment [10,11]. The G8 screening tool is widely used in the oncological setting as a rapid instrument that, across several items—including nutrition, comorbidities, and mobility—provides information on frailty and functional impairment [12]. In addition, handgrip strength measurement is considered a simple, dynamic test of muscle function that can be implemented in both outpatient and inpatient settings with minimal equipment and is discussed as a surrogate marker for frailty, functional reserve, and mobility [13,14,15,16].

Although sarcopenia, frailty, and functional tests are conceptually closely related, data systematically examining the relationship between CT-defined low skeletal muscle mass and simple clinical screening instruments in everyday oncology practice are still lacking. In particular, it remains unclear whether the combination of G8 screening outcomes and handgrip strength is associated with CT-defined low skeletal muscle mass or whether these tools rather capture complementary aspects of frailty-related vulnerability and functional status.

At the same time, nutrition—including food preferences, avoidance of certain foods, use of nutritional supplements, and self-reported adherence to special or cancer-specific diets—is discussed as a potentially modifiable factor in the context of sarcopenia, whereas data on the actual nutritional behaviour of older oncology patients and its association with imaging-based muscle status are limited.

Against this background, we investigated in a cohort of older patients with malignant hematologic neoplasms and solid tumors the relationship among CT-derived L3 skeletal muscle index, CT-defined low skeletal muscle mass according to Prado cut-offs, handgrip strength, G8-based frailty screening results, and self-reported nutritional behavior. The aim of our exploratory cross-sectional analysis was to assess associations among these measures and to evaluate whether they reflect overlapping or complementary dimensions of functional and frailty-related vulnerability. Frailty-related vulnerability was operationalized by the G8 total score and the G8 item C mobility score. Furthermore, we evaluated whether specific nutrition-related behavioral patterns were associated with CT-defined low skeletal muscle mass.

## 2. Materials and Methods

### 2.1. Patients and Study Design

This analysis is based on a previously published, prospectively collected dataset of 300 inpatients treated at the Department of Hematology and Medical Oncology at the University Medical Center Göttingen [17]. The original study was an anonymous, cross-sectional screening study conducted between December 2022 and December 2023, including patients aged ≥ 65 years with an underlying oncological disease, an inpatient stay, and the ability to provide information and informed consent. Participation was offered to all eligible inpatients treated during the predefined one-year recruitment period.

Within the original prospective screening project, standardized data on geriatric status and nutritional behavior were collected for all included patients. The Geriatric 8 (G8) screening tool [18] was used to identify potentially vulnerable older adults who might benefit from a more comprehensive geriatric assessment. In the present analysis, the G8 total score was used to operationalize frailty-related vulnerability, while functional mobility was specifically assessed using G8 item C. G8 item C assesses mobility using three response categories: “bed or chair bound,” “able to get out of bed/chair but does not go out,” and “goes out.” These categories correspond to increasing mobility scores within the G8 framework; therefore, lower G8 item C scores were interpreted as indicating more pronounced mobility limitation within the G8 screening framework. No separate objective mobility test was performed. Handgrip strength was considered an objective functional measure and analyzed in relation to both CT-derived L3-SMI and G8-based frailty screening outcomes. In addition, nutritional behavior was assessed with a structured set of questions [19], as previously described: patients were asked whether they avoided or preferred specific foods, whether they used dietary supplements, and whether they followed a special or cancer-specific diet. For the purposes of this survey, a special or cancer-specific diet was defined as any patient-reported dietary regimen adopted in relation to the cancer diagnosis or cancer treatment; no predefined therapeutic diet was assumed. These four items were used to operationalize cross-sectional self-reported nutritional behavior and were not interpreted as longitudinally assessed dietary changes.

As part of the clinical examination at screening, handgrip strength was measured on the dominant hand in a standardized seated position using a Baseline^®^ Smedley digital hand dynamometer (model 12-0286), and the highest value from repeated trials was used for analysis. Handgrip strength was categorized as low, normal, or high according to age- and sex-adjusted reference ranges provided in the manufacturer’s documentation accompanying the Baseline^®^ Smedley digital hand dynamometer, model 12-0286; Fabrication Enterprises, White Plains, NY, USA.

Because these reference ranges are device-specific manufacturer-provided normative values and do not represent consensus-based diagnostic thresholds for sarcopenia, the exact cut-off values used for classification are reported below. In men, the respective cut-offs were <28.2, 28.2–44.0, and >44.0 kg for patients aged 65–69 years, and <21.3, 21.3–35.1, and >35.1 kg for patients aged ≥70 years. In women, the corresponding cut-offs were <15.4, 15.4–27.2, and >27.2 kg for patients aged 65–69 years, and <14.7, 14.7–24.5, and >24.5 kg for patients aged ≥70 years. These categories were used to describe handgrip strength status relative to device-specific normative values and were not intended to define probable sarcopenia according to EWGSOP2 criteria. Information on hand grip strength was available for 112 of 116 patients.

The study protocol of the original screening project was approved by the Ethics Committee of the University of Göttingen (approval number 26/8/22, approval date: 1 December 2022), and the present analysis was conducted within the framework of this ethical approval. All procedures were carried out in accordance with the principles of the Declaration of Helsinki and current data-protection regulations. This observational study was reported in accordance with the STROBE guideline for cross-sectional studies [20], and the completed checklist is provided in Appendix A.

For the present single-center observational cross-sectional substudy, we retrospectively evaluated the availability of computed tomography (CT) imaging performed within one month before to one month after the date of screening inclusion. CT scans had been acquired as part of routine clinical care and were not performed for study-specific purposes. Among the 300 patients of the original cohort, CT scans with a clearly identifiable L3 level were available for 116 individuals (38.7%), enabling assessment of L3-SMI and CT-defined low skeletal muscle mass. To assess potential selection bias, available baseline characteristics of patients included in the CT-based substudy were descriptively compared with those of patients from the original screening cohort without eligible CT imaging.

Skeletal muscle mass was assessed using computed tomography (CT) images obtained as part of routine clinical care within the predefined time window of one month before to one month after study inclusion. For all patients with available imaging, axial CT slices at the level of the third lumbar vertebra (L3) were identified and analyzed. An experienced physician with expertise in oncological imaging manually contoured the skeletal muscle area at L3, including the psoas, paraspinal, and abdominal wall muscles, using a standardized segmentation approach.

The cross-sectional skeletal muscle area (cm^2^) at L3 was then normalized to patient height to calculate the skeletal muscle index (SMI, cm^2^/m^2^). According to the Prado criteria, low skeletal muscle mass was defined as an SMI < 52.4 cm^2^/m^2^ in men and <38.5 cm^2^/m^2^ in women [21]. Patients with an SMI below these thresholds were classified as having CT-defined low skeletal muscle mass according to Prado cut-offs. Because the present classification was based on muscle quantity derived from CT imaging and did not require concomitant functional impairment, the term CT-defined low skeletal muscle mass is used throughout the manuscript rather than clinical sarcopenia, unless explicitly referring to the broader syndrome.

Potential sources of bias were addressed by using predefined eligibility criteria, standardized assessment procedures, and a consecutive screening approach during the defined recruitment period. Inclusion in the CT-based substudy depended on image availability and technical suitability for L3 assessment, not on functional status, nutritional behavior, or suspected sarcopenia. Statistical methods were selected according to subgroup size and distributional characteristics of the data.

### 2.2. Statistical Analysis

Data were collected in a spreadsheet (Microsoft Excel, Microsoft Office, Version 2021) and subsequently analyzed. Boxplots were generated using Excel. All statistical tests were performed using the web-based software Social Science Statistics (https://www.socscistatistics.com/tests/; last accessed 17 February 2026). Descriptive statistics were used to summarize clinical characteristics. Missing data were not imputed. Analyses were performed using available cases for each variable or comparison, and the corresponding denominators are reported in the respective tables, figures, and result summaries where applicable. All analyses were exploratory and unadjusted. The decision not to perform multivariable regression modeling was made a priori because the CT-based substudy was limited to 116 patients, with further reductions in available cases for some variables, and because the analysis addressed several interrelated exploratory endpoints rather than a single pre-specified primary outcome. Given the limited number of patients within clinically relevant subgroups, the inclusion of multiple covariates such as age, sex, tumor entity, BMI, handgrip strength, G8 score, and nutritional behavior would have resulted in unstable estimates and a high risk of overfitting. As a limited sensitivity approach, key analyses involving SMI were examined in sex-specific strata because SMI distributions and Prado cut-offs are sex-specific. In addition, the prevalence of CT-defined low skeletal muscle mass was compared between patients with hematologic malignancies and solid tumors. Further stratified analyses by additional clinical variables were not performed because subgroup sizes would have become too small to yield interpretable estimates. Therefore, the statistical analysis was restricted to descriptive statistics, univariable group comparisons, and correlation analyses. The results should consequently be interpreted as exploratory associations rather than confounder-adjusted or causal effects. No multivariable regression models were performed; therefore, no confounder-adjusted estimates were calculated and no covariates were selected for adjustment. Owing to occasionally small subgroup sizes, Fisher’s exact test was applied instead of the chi-square test. Spearman’s rank correlation coefficient (Spearman’s rho) was used for correlation analyses. Group comparisons of continuous variables (e.g., SMI and G8 score) were conducted using the Mann–Whitney U test. A two-sided *p*-value < 0.05 was considered statistically significant. Principal component analysis and correlation heat map analysis were performed using MetaboAnalyst (version 6.0, [22]). For nutritional behavior, the four variables were binary self-report items: preference for specific foods, avoidance of specific foods, use of nutritional supplements, and adherence to a special or cancer-specific diet. These items were first analyzed descriptively as frequencies and percentages and compared between patients with and without CT-defined low skeletal muscle mass using Fisher’s exact test. In addition, PCA and correlation heat-map analysis were used as exploratory graphical tools to visualize combined nutritional behavior patterns and pairwise co-occurrence among items. This approach was used descriptively, in line with previous work from our group applying PCA-based visualization to binary patient-reported behavioral variables [23]. To enable log2 transformation of binary variables containing zero values, a small pseudocount of 0.0001 was added before transformation, resulting in log2(x + 0.0001)-transformed values. The transformed values were used only for exploratory graphical visualization and not for inferential modeling. Because of the binary nature and small number of variables, PCA results were not interpreted as evidence of stable latent dimensions. PCA results were visualized using PC1 and PC2 with 95% confidence regions; overall group separation in the PCA space was assessed by PERMANOVA based on 999 permutations. Correlation heat maps were generated in MetaboAnalyst (version 6.0) based on pairwise correlations.

For language editing and stylistic refinement of the manuscript, the authors used ChatGPT, version GPT-5.5 Thinking, developed by OpenAI. The tool was used exclusively to improve readability, grammar, and consistency of wording; it was not used for study design, data collection, statistical analysis, interpretation of results, or generation of scientific conclusions.

## 3. Results

### 3.1. Handgrip Strength Was Not Associated with CT-Defined Low Skeletal Muscle Mass

To assess potential selection bias related to CT availability, patients included in the CT-based substudy were compared descriptively with the 184 patients from the original screening cohort without eligible CT imaging. The two groups were broadly comparable with respect to BMI and malignancy type, whereas the CT-based substudy included a lower proportion of male patients and slightly lower G8 total and item C mobility scores. These differences indicate that representativeness of the CT-available subgroup cannot be assumed (Supplementary Table S1).

Overall, 116 patients were included, of whom 68/116 had malignant hematologic neoplasms (Table 1) and 48/116 had solid tumors. CT-defined low skeletal muscle mass was present in 64/116 patients (55.2%) and was significantly more frequent in men than in women (*p* < 0.01, Fisher’s exact test).

As an exploratory sensitivity comparison, CT-defined low skeletal muscle mass did not differ significantly between patients with malignant hematologic neoplasms and those with solid tumors (*p* > 0.05, Fisher’s exact test).

**Figure 1:**
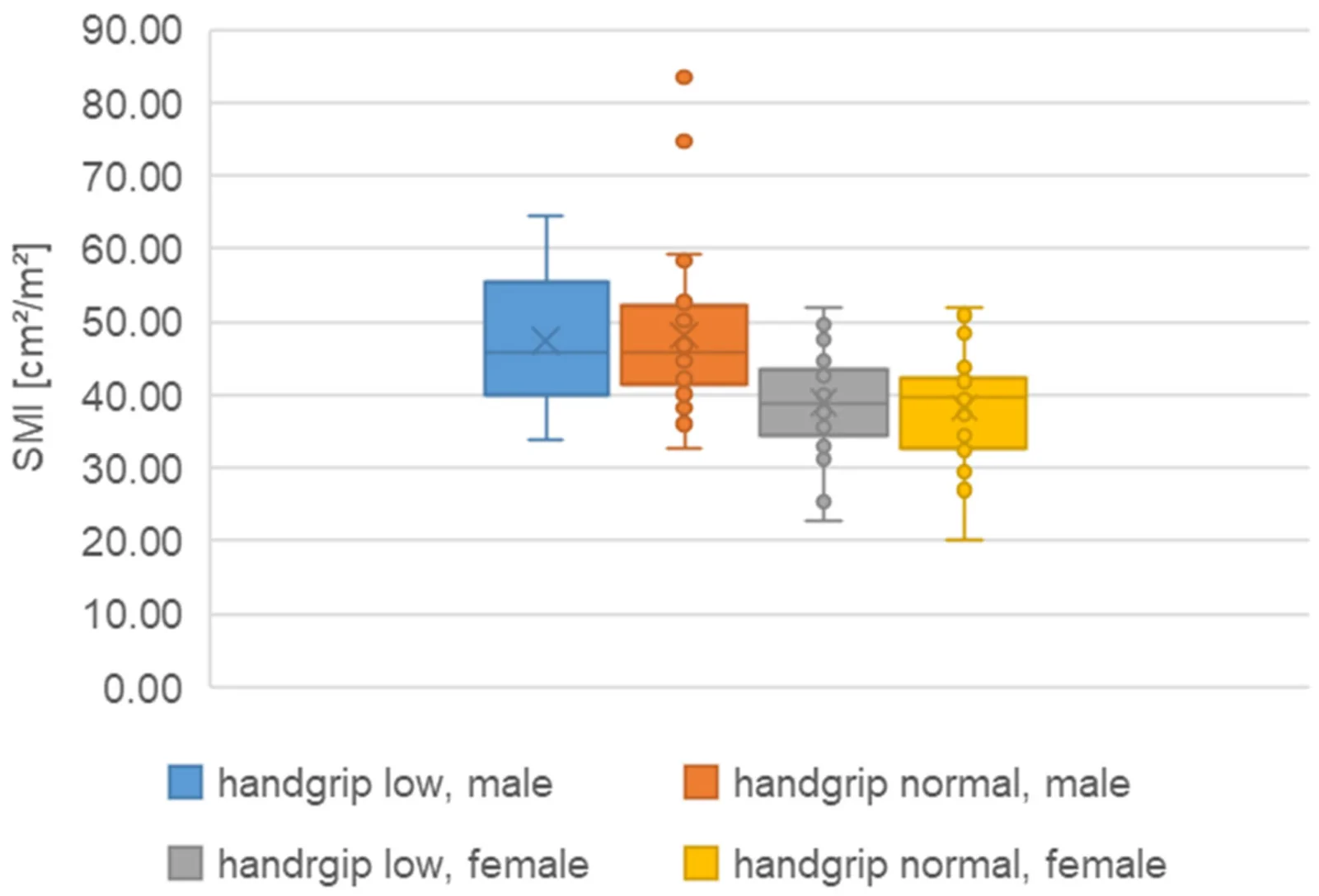
Skeletal muscle index (SMI) according to handgrip strength status and sex. Boxplots show SMI values stratified by low versus normal handgrip strength in male and female patients, reflecting sex-specific SMI distributions and cut-offs. No significant difference in SMI was observed between patients with low and normal handgrip strength within sex-specific groups. Boxes indicate the interquartile range, horizontal lines denote medians, whiskers represent the data range excluding outliers, and individual dots indicate patient-level observations. SMI, skeletal muscle index.

Median SMI values were compared between patients with low and normal handgrip strength. Because sex-specific SMI cut-offs were applied, analyses were performed separately for male and female patients. In male patients, median SMI was 45.68 cm^2^/m^2^ (IQR 39.92–55.44) in the low handgrip strength group and 45.74 cm^2^/m^2^ (IQR 41.48–52.17) in the normal handgrip strength group. In female patients, median SMI was 38.66 cm^2^/m^2^ (IQR 34.54–43.43) and 39.70 cm^2^/m^2^ (IQR 32.57–42.38), respectively. No significant differences were observed between handgrip strength groups within either sex-specific subgroup. Overall, no significant difference between SMI of patients with low and normal handgrip strength was observed (Figure 1). These findings indicate that handgrip strength status was not associated with CT-derived SMI in this cohort. Similarly, no significant association was observed between handgrip strength status and CT-defined low skeletal muscle mass (Spearman’s rho = 0.026; *p* = 0.784).

### 3.2. Low Handgrip Strength Was Associated with Lower G8 Total Scores and Lower G8 Item C Mobility Scores

**Figure 2:**
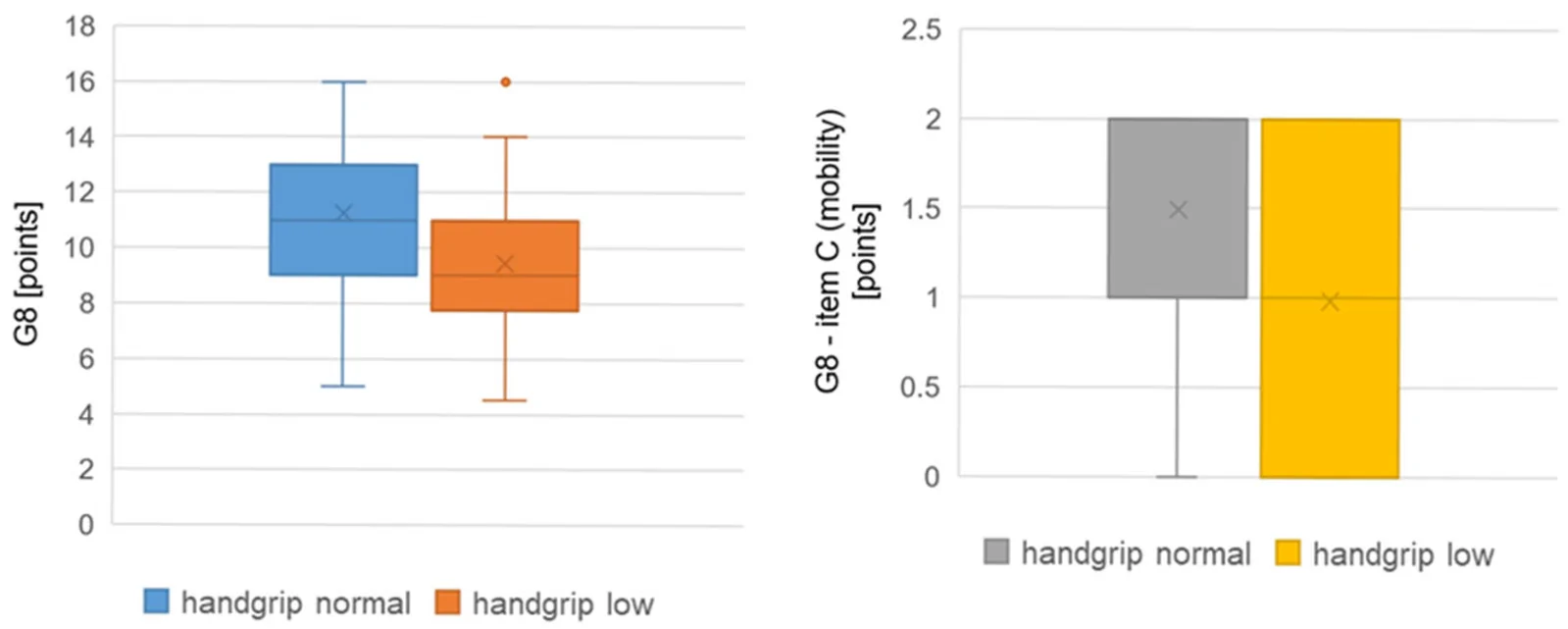
Association of handgrip strength status with frailty screening and mobility. Boxplots compare overall G8 screening scores and G8 item C mobility scores between patients with normal and low handgrip strength. Patients with low handgrip strength showed significantly lower G8 scores and lower mobility scores than patients with normal handgrip strength, indicating greater frailty-related vulnerability and lower mobility scores within the G8 screening framework. Boxes indicate the interquartile range, horizontal lines denote medians, whiskers represent the data range excluding outliers, crosses indicate mean values, and individual dots represent outliers. G8, Geriatric 8 screening tool; item C, mobility item of the G8 questionnaire. *p* < 0.01.

While handgrip strength does not seem to be associated with muscle mass assessed at L3 in CT scans, patients with low handgrip strength had significantly lower overall G8 scores than those with normal handgrip strength (median 9, IQR 7.75–11 vs. median 11, IQR 9–13; *p* < 0.001). Similarly, G8 item C mobility scores were lower in the low handgrip strength group than in the normal handgrip strength group (median 1, IQR 0–2 vs. median 2, IQR 1–2; *p* = 0.001). Handgrip strength status was positively correlated with both the overall G8 score (Spearman’s rho = 0.362; *p* < 0.001) and the G8 item C score (rho = 0.327; *p* < 0.001; Figure 2).

### 3.3. No Association Between CT-Defined Low Skeletal Muscle Mass and Self-Reported Nutritional Behavior

Patient assessments included four nutrition-related behavioral items: preference for specific foods, avoidance of specific foods, use of nutritional supplements, and adherence to a cancer-specific diet. Overall, 14.7% (17/116) patients preferred certain foods, 39.7% (46/116) avoided certain foods, 38.8% (45/116) used nutritional supplements and 7.8% (9/116) reported adherence to a special or cancer-specific diet.

**Figure 3:**
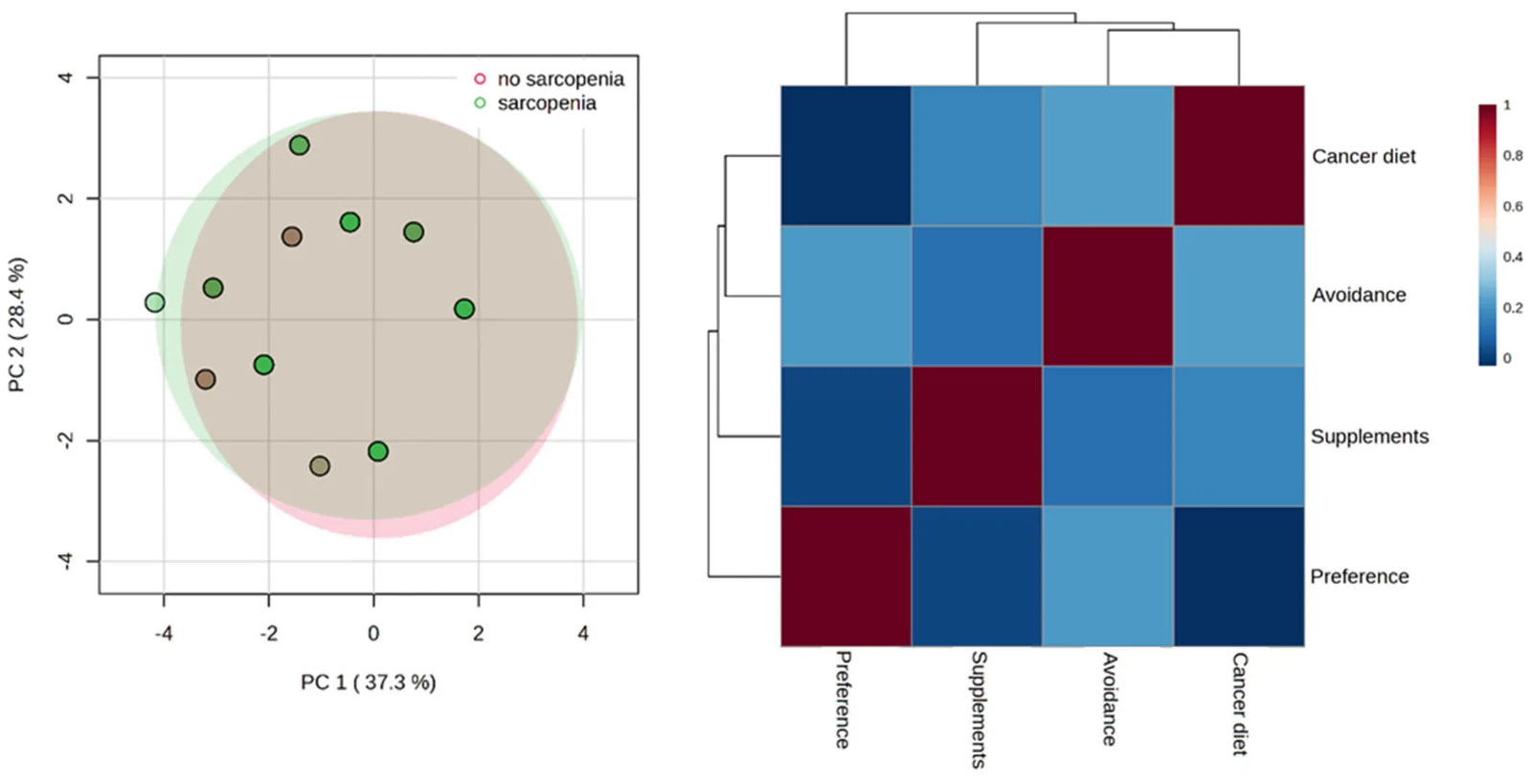
Nutritional behavior patterns according to CT-defined low skeletal muscle mass status. Principal component analysis showed no clear separation between the two groups (patients with and without CT-defined low skeletal muscle mass), indicating comparable patterns of self-reported nutritional behavior across groups. The correlation heatmap with hierarchical clustering illustrates relationships among nutritional behavior items and revealed the following descriptive associations: special/cancer-specific diet–food avoidance, special/cancer-specific diet–supplement use, and supplement use–food avoidance. These findings suggest that nutritional behavior profiles did not differ visibly according to CT-defined low skeletal muscle mass status. These visualizations are exploratory and were not used to infer stable latent behavioral dimensions. These findings suggest that nutritional behavior profiles did not differ visibly according to CT-defined low skeletal muscle mass status. PCA, principal component analysis.

No significant differences in any of these individual behaviors were observed between patients with and without CT-defined low skeletal muscle mass. PCA was used as an exploratory graphical visualization of the combined binary nutritional behavior items and showed no clear separation between patients with and without CT-defined low skeletal muscle mass (Figure 3). The correlation heat map was used descriptively to visualize pairwise co-occurrence among the four items and suggested co-occurrence of special or cancer-specific diet adherence with food avoidance and supplement use, as well as co-occurrence of supplement use with food avoidance.

## 4. Discussion

In the present study, we evaluated handgrip strength as a readily applicable clinical measure in relation to CT-derived muscle status. Handgrip strength can be easily integrated into routine clinical examination, is inexpensive, and does not involve radiation exposure, making it an attractive tool for assessing functional impairment in older oncology patients. However, we observed no difference in SMI between patients with low and normal handgrip strength. This finding contrasts with previous studies reporting associations between skeletal muscle mass and handgrip strength. Meerkerk et al. described a strong association in older patients with head and neck cancer; however, muscle mass was assessed at the C3 rather than the L3 level, which may partly explain the divergent findings [24]. Moreau et al. also reported a correlation between L3-derived SMI and handgrip strength in patients with digestive cancers undergoing chemotherapy [25]. Differences in patient populations, tumor entities, imaging levels, and analytical approaches may therefore contribute to the inconsistent relationship between CT-derived muscle mass and handgrip strength across studies.

At the same time, the literature contains more ambiguous findings suggesting an incongruence between low SMI and handgrip strength. Van Jacobs et al. showed that not all patients with low SMI present with decreased handgrip strength, and vice versa [26]. Another study likewise did not observe an association between SMI and handgrip strength [27]. These results align with our observation. Differences in analytic strategy may further contribute to discrepancies between studies: while some authors compared handgrip strength and SMI as continuous variables, we—similar to van Jacobs et al. and Héctor et al.—first classified patients according to CT-defined low L3-SMI and then compared parameters between groups. In addition, we categorized handgrip strength using age- and sex-specific reference values. Consequently, we did not analyze raw dynamometer values as continuous data, but categorized patients as having normal/high versus low handgrip strength. This categorization may also contribute to divergent results. Importantly, we consider this approach a strength rather than a limitation, as it reflects an individualized interpretation of handgrip strength adapted to age and sex. Other studies have also reported no correlation between handgrip strength and SMI.

Regarding frailty and handgrip strength, we reproduced previous findings showing an association between the G8 score and handgrip strength [24,28]. To our knowledge, we are the first to perform a subanalysis comparing the results of item C (mobility item) with handgrip strength. The G8 describes patients’ functional reserve, and item C specifically reflects mobility in everyday life [29]. Our results indicate that subjectively reported or clinically assessed mobility (item C) is also reflected by the objective parameter handgrip strength. To date, handgrip strength has often been viewed as a parameter that complements the G8 in the context of sarcopenia screening and muscle health assessment, as the G8 is frequently considered a tool focusing on nutrition and vulnerability. However, our data suggest that item C itself already captures functional information appropriately. In line with this, an association between mobility limitation and low handgrip strength has been described previously [30]. Thus, our findings indicate that this established relationship is also detected within the G8, specifically via item C.

Given that handgrip strength reflects functional status and is frequently incorporated into nutritional assessment in oncology, we further asked whether CT-defined low muscle mass is associated with differences in self-reported nutritional behavior [31,32,33]. Using our previously proposed tool to assess nutritional behavior [17,19], we did not observe differences in food preference, food avoidance, supplement use, or adherence to a special or cancer-specific diet between patients with low and normal CT-defined muscle mass in principal component analysis. These findings suggest that the assessed cross-sectional self-reported nutritional behavior items did not differ according to CT-defined low skeletal muscle mass status. They should not be interpreted as evidence regarding longitudinal dietary change or actual nutrient intake. This argues against the intuitive assumption that patients with higher disease burden are more prone to nutritional changes and is consistent with the observation that cancer cachexia and sarcopenia in cancer patients are not primarily mediated by diet changes (i.e., individual decisions/adaptations of diet) but rather represent a multifactorial process [34]. Collectively, these findings support the notion that clinical assessments should not be limited to questions on nutritional behavior alone; assessment of functionality and muscle status should be integrated as well. Moreover, since nutritional behavior did not differ between patients with low and normal CT-defined muscle mass, nutritional counseling and education should be offered to all patients.

Although we did not observe group differences, we assumed that nutrition-related behaviors may still co-occur in clinically relevant patterns within the overall cohort. Therefore, we used correlation heat-mapping to identify associated behaviors among the surveyed patients. Overall, self-reported adherence to a special or cancer-specific diet co-occurred descriptively with supplement use and food avoidance. Recognizing this clustering behavior implies both opportunities (high motivation for change) and risks (restrictive diets and potential risk of malnutrition) in clinical practice, which should be addressed and reflected in nutritional counseling.

From a clinical perspective, these findings support the routine use of handgrip strength as a pragmatic, low-threshold measure to capture functional impairment and frailty-related vulnerability, even when CT-derived L3-SMI does not differ between strength categories. The association between handgrip strength and the G8—especially item C—suggests that mobility information obtained through geriatric screening corresponds to objective functional performance. In parallel, the absence of differences in self-reported nutritional behavior between patients with low and normal CT-defined muscle mass reinforces the need to assess muscle status and functionality beyond nutritional questionnaires alone. Finally, the co-occurrence of cancer diet adherence, supplement use, and food avoidance points to counseling-relevant behavior patterns that should be addressed to prevent unintended dietary restriction. Overall, our results advocate for a multidimensional assessment and counseling pathway in oncologic care.

### Limitations

Several limitations should be acknowledged. First, the sample size of the present substudy was determined by the availability of routine CT imaging suitable for L3 assessment. The analysis was conducted within a previously published, prospectively collected single-center screening cohort of 300 older oncology inpatients [17]. Importantly, CT imaging with an identifiable L3 level was available only for a subset of the original cohort (116/300; 38.7%), and scans were obtained as part of routine clinical care rather than for study-specific purposes. This may introduce selection bias because patients undergoing CT might differ systematically from those without eligible imaging (e.g., in disease burden, staging requirements, or treatment status), and it limits the generalizability of our findings beyond similar inpatient oncology settings. In addition, CT scans were permitted within a predefined time window of one month before to one month after screening inclusion. While this approach reflects real-world availability, temporal variability in clinical status and treatment exposure across this interval may have attenuated associations between CT-derived muscle mass and concurrent functional parameters.

Second, our operational CT-based low-muscle-mass definition relied on L3 SMI and sex-specific cut-offs proposed by Prado et al., which were originally derived from patients with solid tumors of the respiratory and gastrointestinal tracts [21]. Given that our cohort included both malignant hematologic neoplasms and solid tumors, potential misclassification cannot be excluded, and alternative cut-offs or complementary imaging markers (e.g., measures of muscle quality such as myosteatosis) might yield additional insights. Furthermore, skeletal muscle segmentation was performed by manual contouring by an experienced physician using a standardized approach. Although this is common practice, we did not formally assess inter- or intra-rater reliability, and variability in CT acquisition parameters inherent to routine imaging may have influenced absolute measurements.

Third, handgrip strength was analyzed after age- and sex-adjusted categorization (low vs. normal/high), which supports clinical interpretability but reduces granularity compared with analyses using continuous dynamometer values [24,25]. This choice may have decreased sensitivity for detecting small associations with SMI and may partially explain discrepancies with studies using continuous metrics. Finally, nutritional behaviors were assessed by structured self-report items (food preference/avoidance, supplement use, adherence to a special or cancer-specific diet), which are susceptible to recall and social desirability bias and do not quantify actual energy or protein intake. Moreover, the cross-sectional design precludes causal inference and does not allow conclusions on trajectories of muscle loss, frailty, or nutritional behavior over time. Notwithstanding these limitations, the standardized bedside assessment (G8 and handgrip strength) combined with pragmatic, real-world CT-derived muscle quantification provides clinically relevant information on how functional vulnerability and imaging-based CT-derived low skeletal muscle mass relate in older hospitalized oncology patients.

Finally, the statistical analysis was intentionally limited to descriptive statistics, univariable comparisons, and correlation analyses. This approach was chosen because of the limited sample size of the CT-based substudy, missing handgrip strength data in a small number of patients, and the exploratory nature of the research questions. As a result, we cannot exclude residual confounding by age, sex, tumor entity, BMI, disease stage, treatment status, or other clinical factors. Although sex-specific and tumor-group comparisons were performed as limited sensitivity analyses, these cannot replace confounder-adjusted modeling. The observed associations should therefore be interpreted as hypothesis-generating and should be validated in larger cohorts using pre-specified multivariable models.

## 5. Conclusions

In summary, this exploratory cross-sectional substudy suggests that CT-derived L3-SMI and handgrip strength reflect related but not interchangeable aspects of muscle health in older hospitalized oncology patients. While L3-SMI provides an imaging-based estimate of skeletal muscle quantity, handgrip strength was more closely associated with G8-based frailty-related vulnerability and the G8 item C mobility score in this cohort. Self-reported nutritional behavior did not differ according to CT-defined low skeletal muscle mass status. These findings are consistent with the concept that imaging-based muscle assessment, functional testing, and nutritional assessment may provide complementary information, but they should be interpreted as hypothesis-generating because of the observational design and limited sample size. Larger prospective studies are needed to validate these associations and to determine their relevance for clinical outcomes.

## Figures and Tables

**Table 1:** Clinical characteristics.

**Clinical Characteristics**	**n (%) or Mean (±SD)**
Male	52/116 (44.83%)
Female	64/116 (55.17%)
65–74 years	64/116 (55.17%)
75–84 years	45/116 (38.80%)
>85 years	7/116 (6.03%)
**Frailty screening and CT-derived muscle assessment**	
G8 score, mean ± SD	10.31 ± 2.70
G8 item C score, mean ± SD	1.22 ± 0.76
BMI, kg/m^2^, mean ± SD	25.11 ± 4.05
Handgrip low	53/112 (47.32%)
Handgrip normal	55/112 (49.11%)
Handgrip high	4/112 (3.57%)
Low L3-SMI according to Prado cut-offs [21]	64/116 (55.17%)
Normal L3-SMI according to Prado cut-offs [21]	52/116 (44.83%)

## Data Availability

The data supporting the findings of this study are not publicly available due to the sensitive nature of health-related patient data. Anonymized data may be made available by the corresponding author upon reasonable request and subject to applicable ethical and data protection regulations.

## Supplementary Materials

The following supporting information can be downloaded at: https://www.mdpi.com/article/10.3390/nu18213550/s1, Table S1: Comparision of subcohorts.

## Abbreviations

The following abbreviations are used in this manuscript:

**Abbreviation**	**Definition**
CT	Computed tomography
SMI	Skeletal muscle index
L3	Third lumbar vertebra
G8	Geriatric 8 screening tool
BMI	Body mass index
SD	Standard deviation
DEXA	Dual-energy X-ray absorptiometry
PCA	Principal component analysis
PC1	Principal component 1
PC2	Principal component 2
PERMANOVA	Permutational multivariate analysis of variance

## Appendix A

STROBE checklist for cross-sectional studies [19].

	**Item No**	**Recommendation**	
**Title and abstract**	1	(*a*) Indicate the study’s design with a commonly used term in the title or the abstract	√
		(*b*) Provide in the abstract an informative and balanced summary of what was done and what was found	√
**Introduction**			
Background/rationale	2	Explain the scientific background and rationale for the investigation being reported	√
Objectives	3	State specific objectives, including any prespecified hypotheses	√
**Methods**			
Study design	4	Present key elements of study design early in the paper	√
Setting	5	Describe the setting, locations, and relevant dates, including periods of recruitment, exposure, follow-up, and data collection	√
Participants	6	(*a*) Give the eligibility criteria, and the sources and methods of selection of participants	√
Variables	7	Clearly define all outcomes, exposures, predictors, potential confounders, and effect modifiers. Give diagnostic criteria, if applicable	√
Data sources/measurement	8 *	For each variable of interest, give sources of data and details of methods of assessment (measurement). Describe comparability of assessment methods if there is more than one group	√
Bias	9	Describe any efforts to address potential sources of bias	√
Study size	10	Explain how the study size was arrived at	√
Quantitative variables	11	Explain how quantitative variables were handled in the analyses. If applicable, describe which groupings were chosen and why	√
Statistical methods	12	(*a*) Describe all statistical methods, including those used to control for confounding	√
		(*b*) Describe any methods used to examine subgroups and interactions	√
		(*c*) Explain how missing data were addressed	√
		(*d*) If applicable, describe analytical methods taking account of sampling strategy	Not applicable
		(*e*) Describe any sensitivity analyses	Not applicable
**Results**			
Participants	13 *	(a) Report numbers of individuals at each stage of study—eg numbers potentially eligible, examined for eligibility, confirmed eligible, included in the study, completing follow-up, and analysed	√, 116/300 patients had a CT scan available for analysis.
		(b) Give reasons for non-participation at each stage	√
		(c) Consider use of a flow diagram	Not included. The cohort derivation was described in the Materials and Methods Section, with 116 of 300 patients from the original screening cohort included based on availability of CT imaging suitable for L3 assessment.
Descriptive data	14 *	(a) Give characteristics of study participants (eg demographic, clinical, social) and information on exposures and potential confounders	√
		(b) Indicate number of participants with missing data for each variable of interest	√, Handgrip strength data were available for 112 of 116 patients.
Outcome data	15 *	Report numbers of outcome events or summary measures	√
Main results	16	(*a*) Give unadjusted estimates and, if applicable, confounder-adjusted estimates and their precision (eg, 95% confidence interval). Make clear which confounders were adjusted for and why they were included	partly reported/not applicable for adjusted estimates
		(*b*) Report category boundaries when continuous variables were categorized	Reported in the Materials and Methods Section, including age- and sex-specific handgrip strength cut-offs and sex-specific SMI cut-offs for classification of CT-defined low skeletal muscle mass.
		(*c*) If relevant, consider translating estimates of relative risk into absolute risk for a meaningful time period	Not applicable
Other analyses	17	Report other analyses done—eg analyses of subgroups and interactions, and sensitivity analyses	√
**Discussion**			
Key results	18	Summarise key results with reference to study objectives	√
Limitations	19	Discuss limitations of the study, taking into account sources of potential bias or imprecision. Discuss both direction and magnitude of any potential bias	√
Interpretation	20	Give a cautious overall interpretation of results considering objectives, limitations, multiplicity of analyses, results from similar studies, and other relevant evidence	√
Generalisability	21	Discuss the generalisability (external validity) of the study results	√
**Other information**			
Funding	22	Give the source of funding and the role of the funders for the present study and, if applicable, for the original study on which the present article is based	√

* Give information separately for exposed and unexposed groups.
