# Supplementary material for: CT-Derived L3 Skeletal Muscle Index, Handgrip Strength, and Nutritional Behavior as Complementary Dimensions of Frailty in Older Oncology Inpatients: A Cross-Sectional Observational Study
Source: Nutrients. 2026 Oct 28;18(21):3550. doi: 10.3390/nu18213550 (PMC13765116; doi:10.3390/nu18213550)
Supplement: Supplementary file 1 [file nutrients-18-03550-s001.zip › nutrients-4422745-supplementary.pdf]

Supplementary Table S1: Comparision of subcohorts

Characteristic	CT substudy n = 116	No eligible CT n = 184
Age group, 65–74 / 75–84 / ≥85 years	64 / 45 / 7	126 / 52 / 6
Sex, male / female	52 / 64	113 / 71
Malignancy type, hematologic / solid	68 / 48	119 / 65
BMI, kg/m^2, mean ± SD	25.11 ± 4.05	25.62 ± 4.31
G8 total score, median (range)	10 (3–16)	11 (4–18)
G8 item C mobility score, median (range)	1 (0–2)	2 (0–2)
